# Rapid iron loading in a pregnant woman with transfusion-dependent thalassemia after brief cessation of iron chelation therapy

**DOI:** 10.1111/j.1600-0609.2008.01092.x

**Published:** 2008-08

**Authors:** Kallistheni Farmaki, Efstathios Gotsis, Ioanna Tzoumari, Vasilios Berdoukas

**Affiliations:** 1 Thalassaemia Unit, General Hospital of CorinthCorinth, Greece; 2 Euromedica/EnkephalosAthens, Greece

**Keywords:** thalassemia major, pregnancy, transfusion iron load, chelation therapy

## Abstract

In general, in women with transfusion-dependent thalassemia, during pregnancy, iron chelation therapy is ceased. We report a splenectomized patient, who was an excellent complier with chelation therapy, who before embarking on a pregnancy showed no evidence of iron overload, with normal cardiac, thyroid function and glucose metabolism. Laboratory findings showed ferritin 67 μg/L, myocardial T_2_* of 34 ms and liver magnetic resonance imaging (MRI) liver iron concentration of 1 mg/g dry weight. She became pregnant by *in vitro* fertilization in October 2006, delivery occurred in June 2007. She breast fed for 2 months. After 12 months without iron chelation, ferritin was 1583 μg/L. Quantitative MRI showed myocardial T_2_* of 27 ms, that the liver iron concentration had increased to 11.3 mg/g dry weight, indicative of moderate to heavy iron load. This case demonstrates that iron overload can develop rapidly and that physicians caring for patients with transfusion-dependent thalassemia should be particularly alert to any discontinuation of chelation therapy over time.

The marked improvement in survival and reduced morbidity in thalassemia major (TM) allows many patients to parent children (1, 2). For women, in many instances assisted reproduction methods *in vitro* fertilization (IVF) need to be used. In general, as pregnancies in TM patients are usually planned, iron chelation therapy is stopped before the patient enters the IVF programme (3). In the past, the impact of such cessation was not easily assessable except by serum ferritin levels. Liver biopsies, though valuable were relatively traumatic and had low acceptance from patients. With the advent of magnetic resonance imaging (MRI) techniques such assessments are more easily accessible (4). The patient, subject of this report was followed prospectively before and after a pregnancy to determine the impact of withholding chelation therapy for a period of 12 months.

## Patient information

The patient is now 38 yr old. She commenced regular blood transfusion at 10 months of age and desferrioxamine iron chelation therapy at the age of 11 yr. In June 2001 with the availability of the oral chelator deferiprone, she commenced combination therapy with deferiprone at 60–80 mg/kg/d in three doses (25% in the morning, 25% in the afternoon and 50% at night) and desferrioxamine at 30–50 mg/kg/infusion/d. She was always very compliant with her chelation therapy. She did not show any cardiac complications, glucose and thyroid metabolism were normal. She was diagnosed as having primary amenorrhea at 16 yr of age and received hormone replacement therapy. She had osteopenia. In October 2006 with IVF she became pregnant. She delivered in June 2007 and breast fed for 2 months. She had ceased chelation therapy from October 2006 till the end of September 2007. She has given written consent to the anonymous publication of this report.

Liver R2^*^ (reciprocal of T2^*^) is linearly proportional to total iron present in an organ and a calibration equation was derived from the least squares fit of liver R2^*^ data of patients who subsequently underwent liver biopsy vs. linear ion concentration (LIC) determined by the liver biopsies. For liver R2 vs. LIC a non-linear calibration curve was determined and a calibration equation is also available (5). In order to use the calibration equations for liver R2^*^ and R2 the same protocols for determining R2 and R2^*^ must of course be used. Our R2^*^ data were obtained with identical protocols as described from a Los Angeles group (6) but a different pulse sequence was used for determining R2, thus our R2 data can only be correlated to LIC in a qualitative way. For the period of the pregnancy all quantitative determinations are based on our R2^*^ data.

Table 1 shows the mean ferritin levels, the red cell consumption, left ventricular ejection fraction (LVEF) as assessed by echocardiography, cardiac T2^*^ and liver iron concentration. The initial and final LIC were determined from the MRI data were estimated to be 1.0 and 11.3 mg/g dry weight, respectively, indicative of normal and moderate to heavy iron load, respectively. The MRI-derived LVEF at the end of the pregnancy was 63%, indicating marginal normal function.

**Table 1:** Iron load parameters over time

Year	Annual mean ferritin	RCC	LVEF	T2 heart	T2* heart	LIC mg/g dw
2001	1500	230	77%	35.5		
2002	126	251	66%	39.6		
2003	92	210	69%	32.8	35.4	1
2004	105	229	67%	38.0	35.3	1.3
2005	142	257	61%	37.8	34.4	1.2
2006	67	242	63%	38.3	34.1	1
2007	1481	230	65%	33.8	27.3	11.3

RCC is the red cell consumption per annum in mL/kg.

LIC is expressed as mg/g dry weight and was assessed by magnetic resonance imaging T2 and T2^*^.

All T values are in milliseconds.

LVEF, left ventricular ejection fraction; LIC, liver iron concentration.

The most striking feature of her results was the rise in the mean ferritin from 46 μg/L, just before the pregnancy to 1583 μg/L after delivery. Liver R2^*^ rose from 1.3 mg/g dry weight to 11.3 mg/g dry weight and cardiac T2* fell mildly from 34.1 to 27.3 ms (albeit still within the normal range but with a downward trend). Even though her LVEF as assessed by MRI remained normal, it is at the lower limit of normal. She was transfused 33 times during the 9 months receiving a total of 11 417 mL of packed red blood cells. The packed red blood cells given, apart from the anticoagulant solution, are enriched with 100 mL of mannitol additive solution after plasma separation in order to prolong red blood cells conservation to 42 d instead of 35 d. Each unit is about 300–350 mL instead of 200–250 mL. The mannitol additive solution (3300 mL for the 33 units) containing only glucose is not regarded as an iron source. The 11 417 mL of packed cells given are equivalent to 8117 mL of conventional red blood cells and would translate to 6.5 g of iron input. Even though the mean pretransfusion Hb level was maintained at 10.8 g/dL her red cell consumption remained unchanged because she had gained 10 kg weight. The total amount of iron received during the pregnancy was higher than usual owing to her weight gain. The liver iron concentration before combination therapy had shown a R2 value >50/s (indicative of moderate to heavy hepatic iron loading) and by 2006 it had improved to 29.4/s.

The liver during the 12-month chelation-free period accumulated 10.3 mg/g dry weight, i.e. 0.85 mg/g dry weight per month. This is equivalent to an increased total body iron load of 109 mg/kg and is compatible with the 6.5 g of iron she received through transfusion during the period without chelation therapy.

## Discussion

This case report demonstrates the iron loading that occurred in one individual patient after the usual practice of cessation of iron chelation therapy for pregnancy. It would be valuable to have results from more patients. It would also seem that the policy of ceasing chelation therapy during the whole of pregnancy may be inadvisable and may be placing patients at risk of increased morbidity and ultimately mortality, especially during a period in which their cardiac function may need to be at its best. In the experience of one of the authors (VB), armed with the understanding that desferrioxamine is not teratogenic, recommencing regular chelation therapy with desferrioxamine after the 16th week of gestation did not result in any fetal abnormalities. In addition, a number of patients who became pregnant unexpectedly and did not stop desferrioxamine therapy until they were aware of the pregnancy had normal outcomes to their offspring. A case report of a patient who received desferrioxamine chelation therapy and gave birth to a normal child is also indicative of the safety of its use during pregnancy (7).

It is also likely that the free iron that results from the iron overload may be very avidly taken up in the liver and possibly the heart (8).

## Conclusion

This case report shows clearly that pregnant women with TM should be monitored carefully for iron loading before they embark upon a pregnancy and afterwards and consideration should be given to offering desferrioxamine chelation therapy after the middle of the second trimester. The main message from this patient is that in some transfusion-dependent patients, cessation of chelation therapy allows rapid iron overload. Patients should be made aware of this risk in order to encourage them to maintain their chelation therapy consistently.

## References

[1] Jensen CE, Tuck SM, Wonke B (1995). Fertility in beta thalassaemia major: a report of 16 pregnancies, preconceptual evaluation and a review of the literature. Br J Obstet Gynaecol.

[2] Skordis N, Christou S, Koliou M, Pavlides N, Angastiniotis M (1998). Fertility in female patients with thalassemia. J Pediatr Endocrinol Metab.

[3] Karagiorga-Lagana M (1998). Fertility in thalassemia: the Greek experience. J Pediatr Endocrinol Metab.

[4] Anderson LJ, Holden S, Davis B (2001). Cardiovascular T2^*^ magnetic resonance for the early diagnosis of myocardial iron overload. Eur Heart J.

[5] St Pierre T, Clark P, Chua-anusorn W (2005). Noninvasive measurement and imaging of liver iron concentrations using proton magnetic resonance. Blood.

[6] Wood JC, Enriquez C, Ghugre N, Tyzka JM, Carson S, Nelson MD, Coates T (2005). MRI R2 and R2^*^ mapping accurately estimates hepatic iron concentration in transfusion-dependent thalassemia and sickle-cell disease patients. Blood.

[7] Singer ST, Vichinsky EP (1999). Deferoxamine treatment during pregnancy: is it harmful?. Am J Hematol.

[8] Sohn YS, Breuer W, Munnich A, Cabantchik ZI (2008). Redistribution of accumulated cell iron: a modality of chelation with therapeutic implications. Blood.

